# Supplementary material for: RNA editing derived epitopes function as cancer antigens to elicit immune responses
Source: Nat Commun. 2018 Sep 25;9:3919. doi: 10.1038/s41467-018-06405-9 (PMC6156571; doi:10.1038/s41467-018-06405-9)
Supplement: Supplementary file 1 — Supplementary Information [file 41467_2018_6405_MOESM1_ESM.pdf]

RNA editing derived epitopes function as cancer antigens to elicit immune responses

Zhang and Fritsche et al.

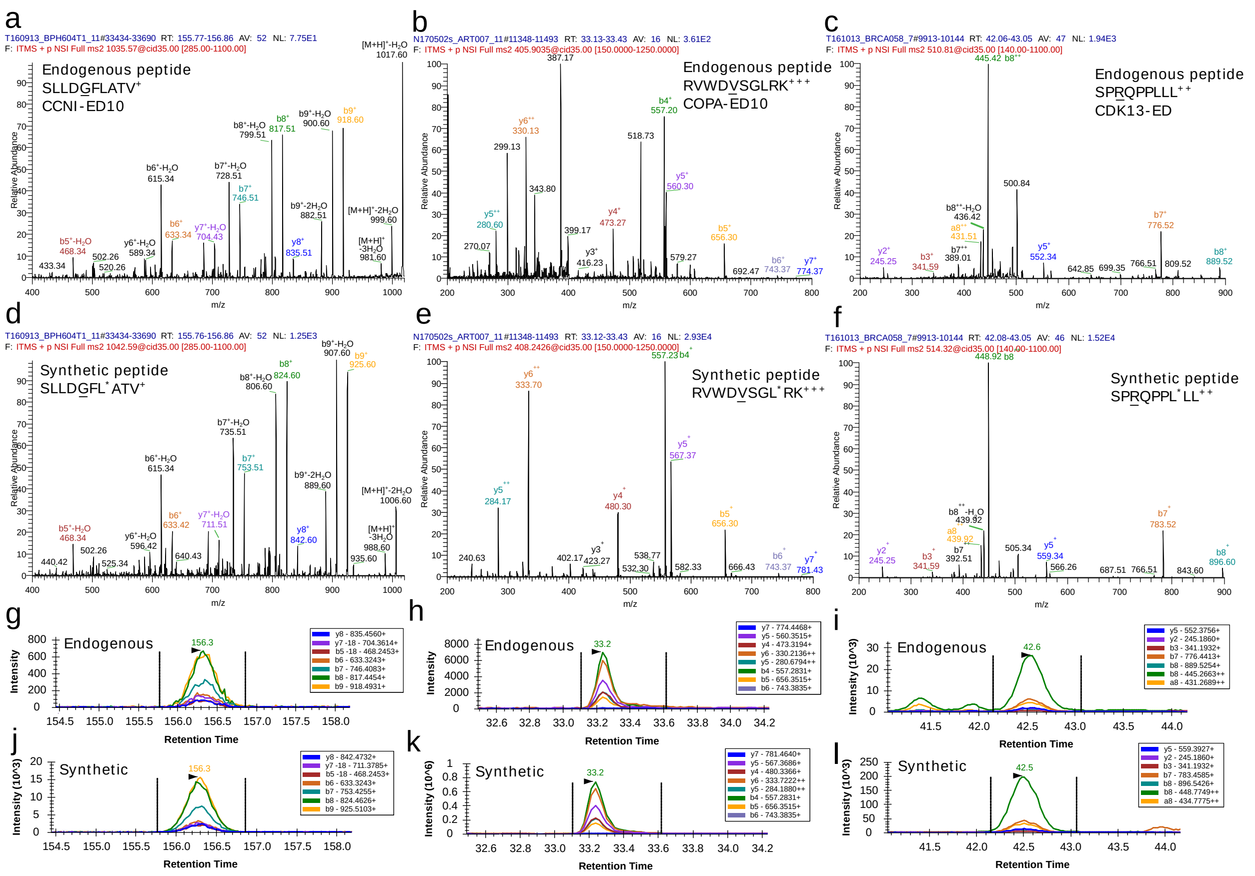

Supplementary Figure 1. LC-MS/MS validation of edited peptide sequence identity based on identical MS/MS spectrum and coelution of synthetic reference. The Fragmentation pattern and retention time of endogenous peptides eluted from tumour sample for singly charged endogenous CCNI-ED10 (**a, g**), triply charged COPA-ED10 (**b, h**) and doubly charged CDK13-ED (**c, i**). Synthetic reference peptides were isotopically labelled at Leucine allowing differentiation between the heavy form (L*) and the light form (L). Peptides labelled either at position seven (CCNI-ED10, CDK13-ED) or eight (COPA-ED10) were spiked into the same tumour sample and are expected to coelute due to identical physicochemical properties if the sequences are identical. Fragments carrying the label show a 7 Da mass shift. Matching fragmentation pattern and retention times for CCNI-ED10 (**d, j**), COPA-ED10 (**e, k**) and CDK13-ED (**f, l**) prove sequence identity.

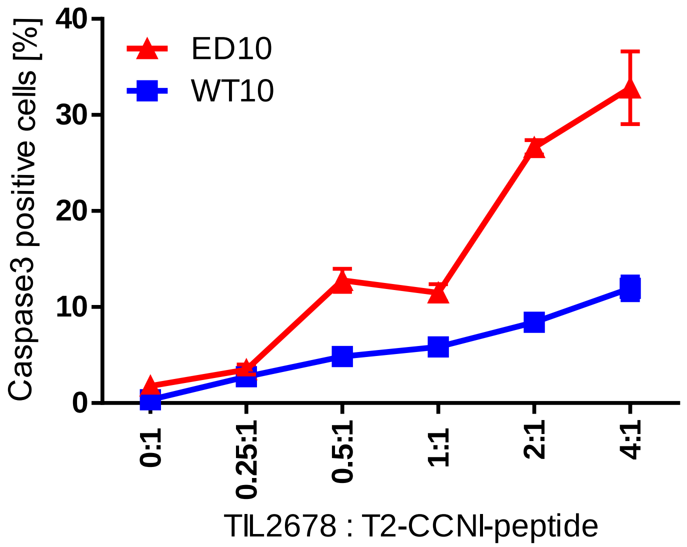

Supplementary Figure 2. Caspase3 based cytotoxic T Lymphocyte (CTL) killing assay. TIL2678 mediated killing of T2 cells pulsed with CCNI-ED10 and CCNI-WT10 is shown. For each condition arithmetic mean±SEM (*n*=3) is reported.

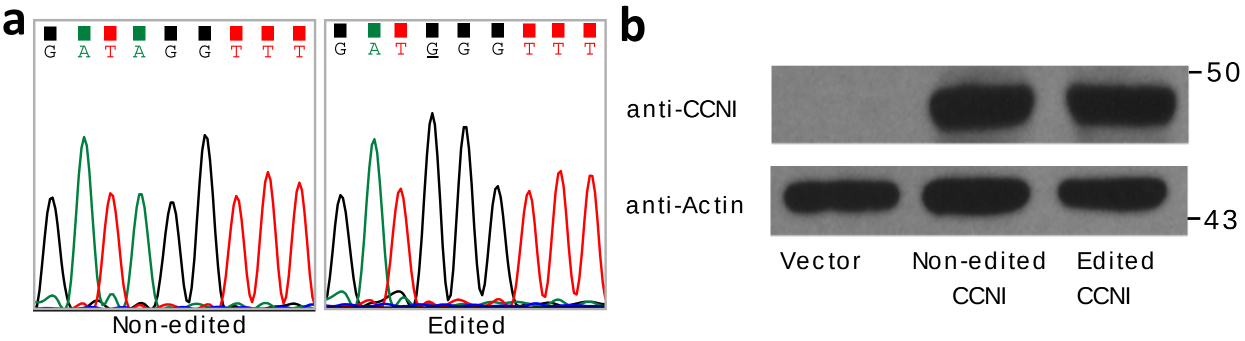

Supplementary Figure 3. Confirm CCNI wildtype and edited gene and protein expression. *CCNI* wildtype and edited genes were cloned into pHAGE vector by Gateway cloning system and transiently transfected into 293-A2 cells. After transfection, total RNA was isolated, and RT-PCR was performed to amplify *CCNI* mRNA using the primers that flanked the *CCNI R75G* editing site. This was followed by PCR product purification and sequencing to confirm wildtype and edited cDNA expression. **a,** *CCNI R75G* editing in the DNA construct was confirmed by PCR and sequencing. The triplet AGG in wildtype was changed to GGG in edited cDNA. **b,** CCNI protein over expression in transfected 293-A2 cells were confirmed by immunoblotting blotting.

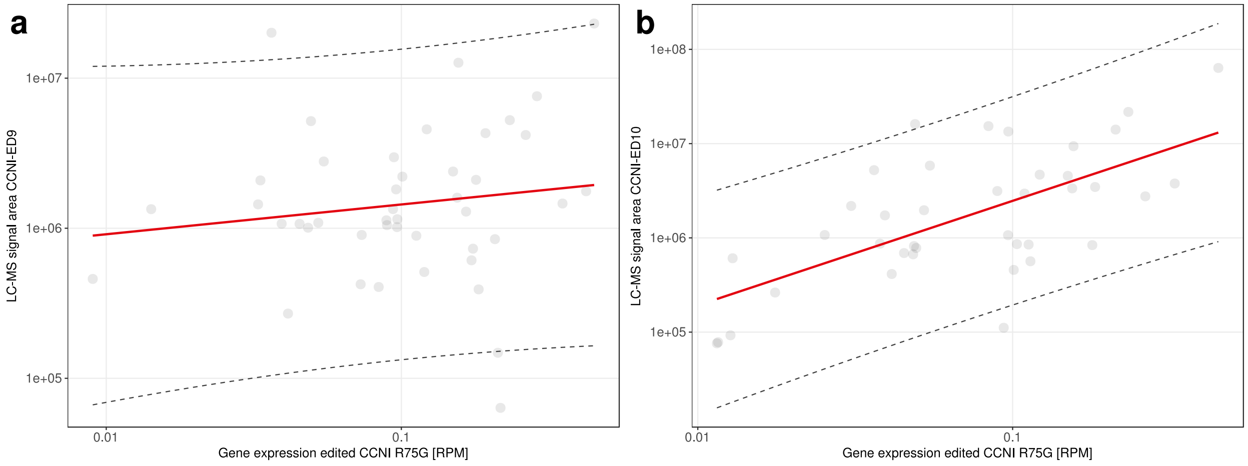

Supplementary Figure 4. Peptide-mRNA correlation. Correlation between gene expression and peptide presentation for CCNI-ED positive samples. The scatterplot includes the regression curve (red line) as well as the 95% prediction interval (dashed lines). CCNI-ED9 (a) shows a correlation of *R*=0.33 (*n*=44, *CI*=0.03-0.57) and CCNI-ED10 (b) of *R*=0.67 (*n*=39, *CI*=0.45-0.81).

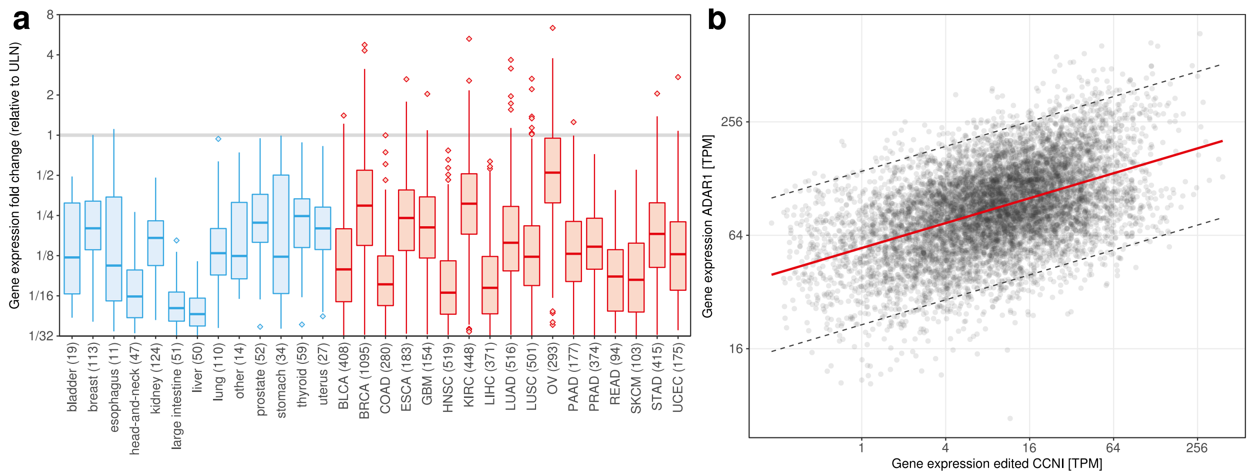

Supplementary Figure 5. TCGA data analysis. a, Gene expression of edited *CCNI* mRNA in TCGA tumours against normal tissues. The number of samples per group is given in parentheses. Normalized read counts were expressed as fold change relative to the upper limit of normal (grey line). The distribution of fold changes is displayed as box plot scaled by sample size and highlighting outliers as diamonds. **b,** Correlation of gene expression between *ADAR1* and edited *CCNI* for TCGA tumours (R=0.48, n=8,241). The scatterplot includes the regression curve (red line) as well as the 95% prediction interval (dashed lines).

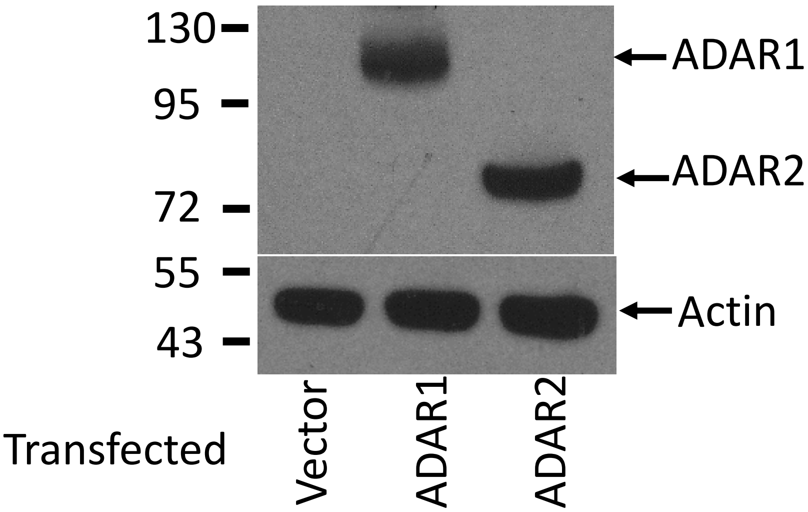

Supplementary Figure 6. Western blotting confirms *ADAR1* and *ADAR2* expression. Human *ADAR1* or *ADAR2* genes tagged by V5 at the C-terminal were cloned into pHAGE vector and transfected to 293T cells. The over-expression of both proteins was confirmed by immunoblotting blotting using anti-V5 antibody. The house keeping protein expression was used as loading control.

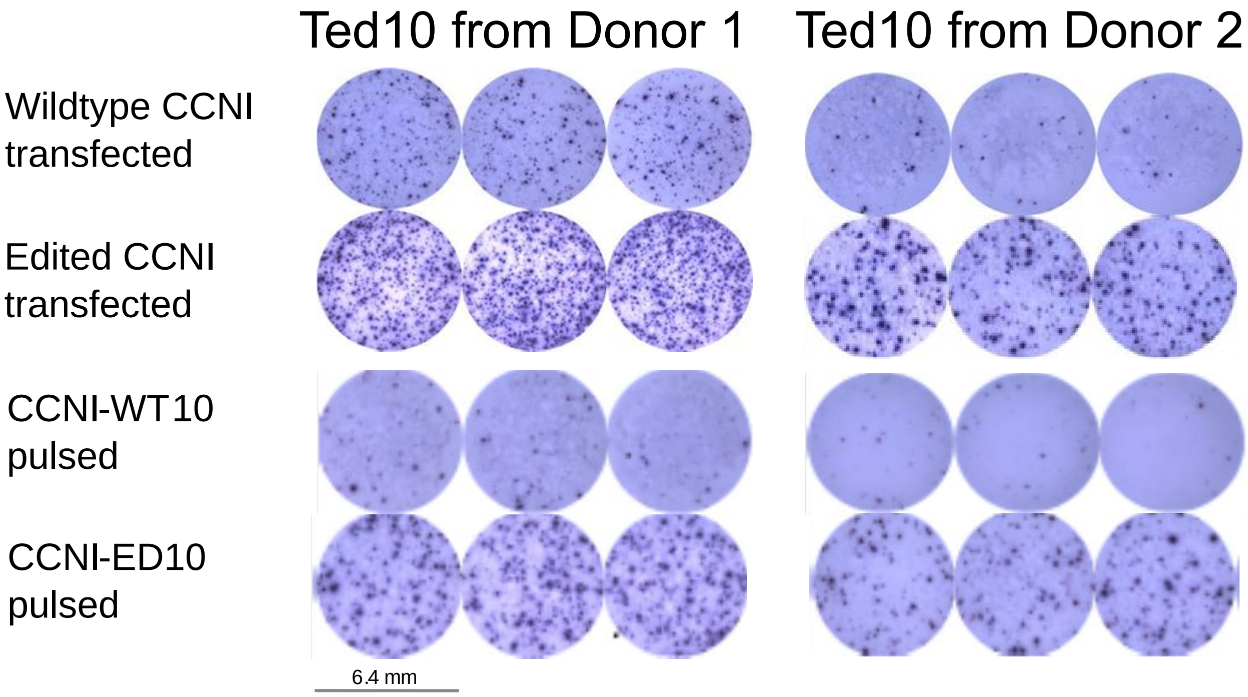

Supplementary Figure 7. CCNI-ED specific T cells are generated using PBMC from two healthy donors. ELISPOT assay showing that both Ted cells can be activated by 293-A2 cells transfected with *CCNI* edited gene or pulsed with peptides to produce IFNɣ.

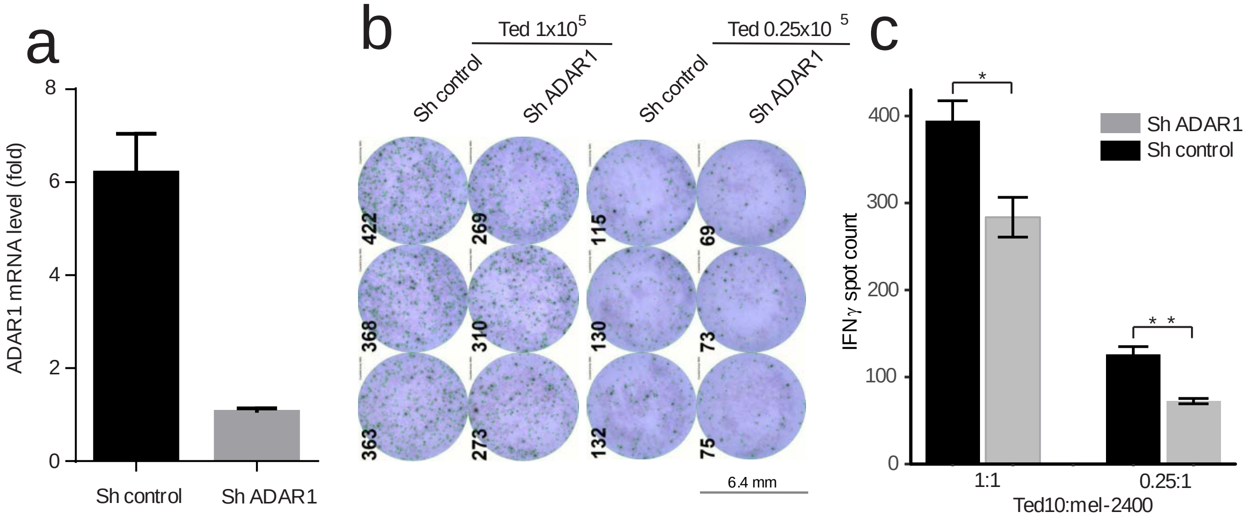

Supplementary Figure 8. Down regulation of *ADAR1* reduces response of mel-2400 to Ted10 T cells. ELISPOT assay showing that knockdown of *ADAR1* mRNA in CCNI-ED10 positive mel-2400 reduced its ability to activate Ted10 cells to produce IFNγ. To knock down *ADAR1* mRNA, Mel-2400 cells were stably transduced with Lentivirus that was made of either empty Lentiviral vector (GFP) control (Sh control) or shRNA *ADAR1* knock-down construct (Sh ADAR1). Cells were sorted based on GFP expression to isolate transduction positive cells. **a,** *ADAR1* mRNA was greatly reduced in Sh ADAR1 expressing mel-2400 compared with the control cells determined by quantitative RT-PCR and normalized to housekeeping gene GAPDH. Mean fold change and standard error of the mean (SEM) is shown for three independent PCR reactions. To determine whether *ADAR1* knock down reduce *CCNI* editing, we cloned *CCNI* PCR product that covers the R/G site into TA cloning vector and picked up 96 bacteria clones for sequencing. We identified 5 edited *CCNI* out of 96 clones from mel-2400 transduced with Sh control, but only 1 edited *CCNI* clone from mel-2400 transduced with Sh ADAR1. **b,** ELISPOT assay showing the reduced response of Ted10 to mel-2400 cells after knockdown of *ADAR1*. 1×10^5 Sh ADAR1 stably expressed mel-2400 cells or control cells were incubated with 1×10^5 of Ted10 (left panel) or 0.25×10^5 of Ted10 (right penal) in triplicate for 18 hours and activated Ted10 cells were measured by ELISPOT assay to detect IFNγ production. Knockdown of *ADAR1* in mel2400 greatly reduced its ability to stimulate Ted10 cell to produce IFNγ. For 1:1 ratio of Ted10 to mel-2400, the mean spot number is 384:284 (p=0.012). For 0.5:1 ratio of Ted10 to mel2400, the mean spots number is 126:72 (p=0.024). **c,** Summary graphs of b. The data was analysed by unpaired T test. For each condition arithmetic mean±SEM (n=3) is reported.

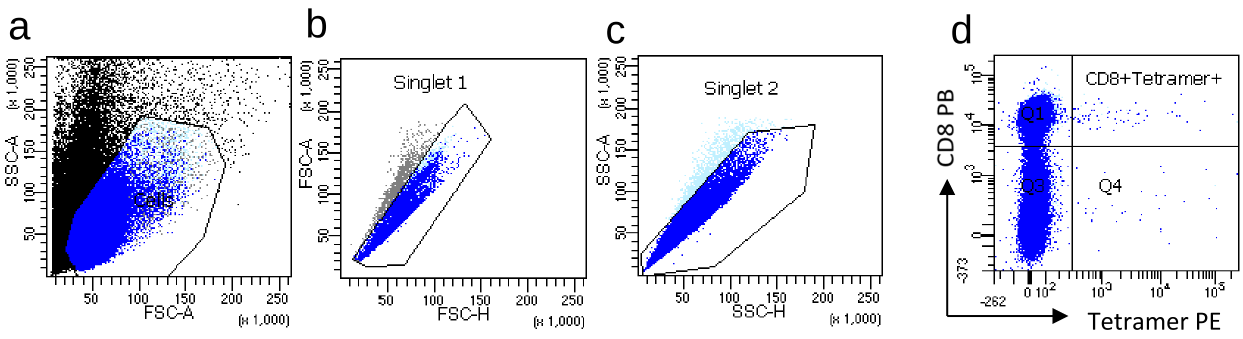

Supplementary Figure 9. Gating strategies used for flow cytometric analysis of CCNI tetramer and CD8 double positive T cell sorting. **a.** Gated on all survival cells. **b.** Gated on all live cells by size. **c**. further separation of single cells. **d.** Tetramer (PE-labeled) and CD8 (pacific blue-labeled) double positive T cells were sorted.

	RNA-seq		LC-MS evidence				
**Editing site**	**#RNA-seq**	**Editing level %**	**FDR %**	**Peptide ion**	**#LC-MS**	**Donor HLA restriction**	**Peptide-spectrum matching**
**COPA I164V**	245	16.2	0.07	RVWDVSGLRK^{+3}	13	A*03:01	successful
			0.07	RVWDVSGLRKK^{+3}	6	A*03:01	successful
**CCNI R75G**	252	5.1	2.40	LLDGFLATV^{+2}	46	A*02:01	successful
			0.56	SLLDGFLATV^{+1}	44	A*02:01	successful
			0.19	SLLDGFLATV^{+2}	6	A*02:01	successful
**HSPA1L K541E**	2	0	0.33	AENALESYAFN^{+2}	2	B*44:03	AENAEFMRNF^{+2}
**FRRS1 R295G**	17	0	0.43	GLADGVM*QCSF^{+2} (M* oxidized)	2	A*02:06	AVADDFC*KV^{+2} (C* w/ Cys-Gly adduct)
**CDK13 Q35R**	20	8.8	0.96	SPRQPPLLL^{+2}	24	B*07:02	successful

Supplementary Table 1: Editing sites identified by LC-MS at 1% false discovery rate (FDR) and supporting RNA and LC-MS evidence. Number of RNA-seq samples with at least 10 reads at the giving site are listed and used to compute average editing level (%). Peptide-level FDR was filtered at 5% FDR to include all different length variants and charge states for a given site. Peptide sequence, charge state and modifications are listed. One of the seven peptides was identified by two peptide ions (singly and doubly charged). Number of identified LC-MS samples at 5% FDR are listed along with the most frequent donor HLA typing. If the suggested peptide sequence did not match the MS/MS spectrum, the correct spectrum interpretation was given. *HSPA1L* editing was ruled out because RNA-seq evidence was missing, the C-terminal amino acid was unusual for B*44:03 binding and the unedited peptide AENAEFMRNF from heme oxygenase 1 was a better match on spectral and HLA level. Wrong sequence assignment was due to precursor mass interference and low fragment mass resolution. *FRRS1* editing also failed validation since there was also no RNA evidence, the sequence did not match the A*02:06 binding motif and the Cys-Gly modified peptide AVADDFCKV from echinoderm microtubule associated protein was a better match and synthetically verified. The cysteine of this peptide can be modified by Cysteinylation or Cysteinylglycine (Cys-Gly). These modifications were not included in the database search and therefore missed.
