# Supplementary material for: RNA editing derived epitopes function as cancer antigens to elicit immune responses
Source: Nat Commun. 2018 Sep 25;9:3919. doi: 10.1038/s41467-018-06405-9 (PMC6156571; doi:10.1038/s41467-018-06405-9)
Supplement: Supplementary file 3 — Description of Additional Supplementary Files [file 41467_2018_6405_MOESM3_ESM.pdf]

Description of Additional Supplementary Files

File Name: Supplementary Data 1
Description: RNA editome peptide database used in screening for HLA-bound peptides. This database contains a total of 2,516 entries for 1,387 edited peptides and their WT counterparts which are derived from 1,369 unique RNA editing sites. Each edited site is flanked by 10 amino acids according to the corresponding protein sequence.
